# Sexual dimorphism following in vitro ischemia in the response to neurosteroids and mechanisms of injury

**DOI:** 10.1186/s12868-020-0553-1

**Published:** 2020-01-29

**Authors:** Raeed Altaee, Claire L. Gibson

**Affiliations:** 1 Department of Neuroscience, Psychology & Behaviour, University of Leicester, Leicester, LE1 9HN UK; 2 Department of Physiology and Pharmacology, University of Karbala, Karbala, Iraq; 3 School of Psychology, University of Nottingham, University Park, Nottingham, NG7 2UH UK

**Keywords:** Ischemia, Sex, Steroid hormones, Neuroprotection, Apoptosis

## Abstract

**Background:**

Cerebral ischemic stroke is a significant cause of morbidity and mortality. Sex differences exist following stroke in terms of incidence, symptoms, outcomes and response to some treatments. Importantly, molecular mechanisms of injury, activated following ischemia may differ between the sexes and if so may account, at least in part, for sex differences seen in treatment response. Here we aimed to determine, using single-sex organotypic hippocampal slice cultures, whether the effectiveness of a potential treatment option, i.e. sex steroids, exhibited any sexual dimorphism and whether sex affected the mechanisms of apoptosis activated following ischemia.

**Results:**

Following exposure to ischemia, male-derived tissue exhibited higher levels of cell death than female-derived tissue. Various sex steroid hormones, i.e. progesterone, allopregnanolone, and estradiol, were protective in terms of reducing the amount of cell death in male- and female-derived tissue whereas medoxyprogesterone acetate (MPA) was only protective in female-derived tissue. The protective effect of progesterone was abolished in the presence of finasteride, a 5α-reductase inhibitor, suggesting it was largely mediated via its conversion to allopregnanolone. To test the hypothesis that sex differences exist in the activation of specific elements of the apoptotic pathway activated following ischemia we administered Q-VD-OPH, a caspase inhibitor, or PJ34, an inhibitor of poly (ADP ribose) polymerase (PARP). Caspase inhibition was only effective, in terms of reducing cell death, in female-derived tissue, whereas PARP inhibition was only protective in male-derived tissue. However, in both sexes, the protective effects of progesterone and estradiol were not observed in the presence of either caspase or PARP inhibition.

**Conclusions:**

Sex differences exist in both the amount of cell death produced and those elements of the cell death pathway activated following an ischemic insult. There are also some sex differences in the effectiveness of steroid hormones to provide neuroprotection following an ischemic insult—namely MPA was only protective in female-derived tissue. This adds further support to the notion sex is an important factor to consider when investigating future drug targets for CNS disorders, such as ischemic stroke.

## Background

Cerebral ischemic stroke is a major cause of mortality and morbidity with limited effective treatments available [1]. Multiple factors influence both the incidence and outcome of ischemic stroke including sex, age, race/ethnicity, hypertension, cardiac disease, diabetes mellitus, hypercholesterolemia, cigarette smoking and alcohol abuse [2]. Sex differences are reported to occur in the causes, symptoms and outcomes following stroke [3]. For example, over the lifespan, women have a higher risk of stroke and increased rates of post-stroke mortality, disability, depression and dementia, compared to men [4]. Such an increased risk and worsened post-stroke seen in women may be a consequence of women’s longer life expectancy due to age being the strongest independent risk factor for stroke [5] and a negative predictor for clinical outcome [6]. However, a significant reduction in the occurrence of stroke and a relatively better outcome following stroke occurs in pre-menopausal women compared to men of the same age [7]. During the menopausal period, females experience a rapid increase in the incidence of stroke compared to males, which is coincident with decreasing levels of the circulating sex hormones, i.e. oestrogens and progesterone [8]. Steroid hormones have been investigated, and demonstrated to be protective, following ischemic stroke using both in vitro and in vivo models [9–12]. However, sex differences may occur in response to treatment, such as steroid hormones, which has been reported previously for aspirin, warfarin and thrombolytic therapy following stroke [13–15].

Ischemic stroke initiates a complex pathology including excitotoxicity, cell necrosis, apoptosis, inflammation, increased oxidative stress and breakdown of the blood brain barrier along with the potential for reperfusion injury [16]. Sex-specific cultures, derived from neonatal populations, demonstrate that female-derived cells are more resistant to ischemic stroke than male-derived cells and following ischemic injury several molecular mechanisms of the injury mechanisms, such as inflammation, cell death, oxidative stress, and microglial activation may function dimorphically [17–19]. The mechanisms of injury following stroke may be affected by sex either as a consequence of intrinsic, i.e. chromosomal, or hormonal differences between the sexes. Sex differences in cerebral ischemia are reported in studies using both neonatal and adult animals suggesting that primary sex hormones are not the only factor influencing sex-influenced neuronal injury [20]. It is likely that sexual dimorphisms become established during development, when hormone levels are low, as a result of variations in cell signalling and response to ischemia [21].

There are important differences between the sexes in the cell death pathways activated following ischemia. For example, it has been demonstrated that female-derived tissue is more sensitive to caspase-mediated cell death, whereas cell death in male-derived tissue is more likely to be triggered by caspase-independent pathways involving the activation of poly (ADP ribose) polymerase (PARP) and the translocation of apoptosis-inducing factor (AIF) [22–24]. Our aim here is twofold—firstly we aim to determine if sex-specific effects occur in the protectiveness of steroid hormones under ischemic conditions and secondly to investigate whether sex affects the mechanisms of cell death activated following ischemia. Improving our understanding of the mechanisms that may underlie sex differences, both in terms of responsiveness to treatment and activated injury mechanisms, may lead to new individualised treatment strategies for disorders such as ischemic stroke.

## Results

**Fig. 1:**
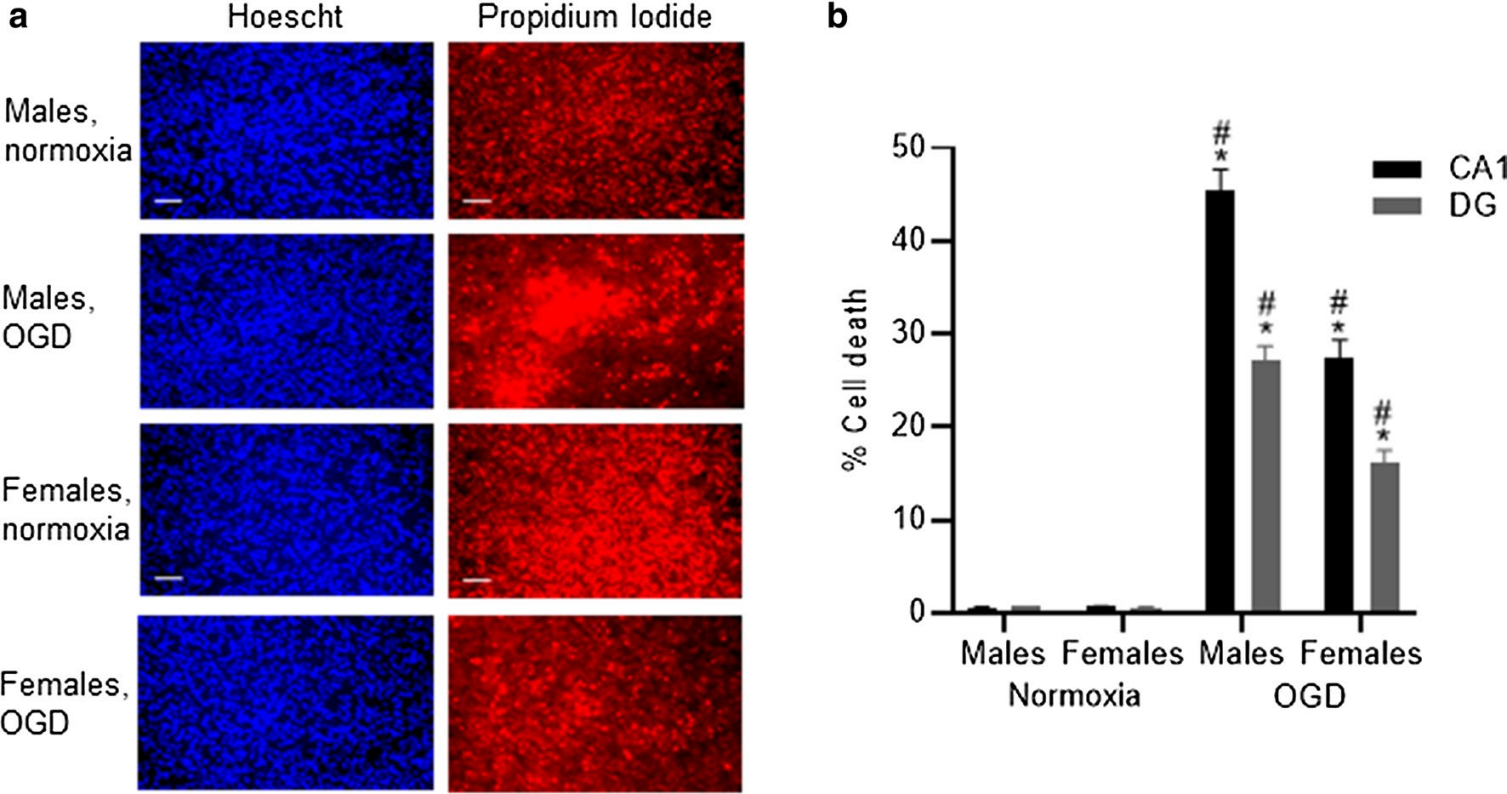
Representative images of hippocampal CA1 slices exposed to normoxic and OGD conditions from male and female animals (**a**). All cell nuclei are shown by Hoechst staining and cell death is shown in images stained with PI (**a**). Cell death was analysed for both the CA1 and dentate gyrus (DG) regions and was found to be significantly increased following exposure to OGD (**b**, *P < 0.001 vs. normoxic control). In addition, following OGD, the amount of cell death seen in male-derived slices was significantly increased compared to female animals in both CA1 and DG regions (# P < 0.001). Data are expressed as mean ± SEM and n = 8 independent wells. Scale bars represent 20 µm

Following oxygen and glucose deprivation (OGD) there was a significant increase in the amount of cell death in both CA1 and dentate gyrus (DG) regions within the neonatal hippocampal slice cultures which was seen in both male (P < 0.001) and female-derived (P < 0.001) slices compared to normoxic controls. Figure 1a shows representative images from the CA1 region of Hoechst and propidium iodide labelled cells for male and female-derived cultures exposed to normoxia and 4 h of OGD. There was a significantly increased amount of cell death in male-derived compared to female-derived slices in the CA1 (45.36 ± 2.34% vs. 27.36 ± 2.10% P < 0.001) and DG (27.25 ± 1.46% vs. 16.13 ± 1.38% P < 0.001) regions (Fig. 1b).

**Fig. 2:**
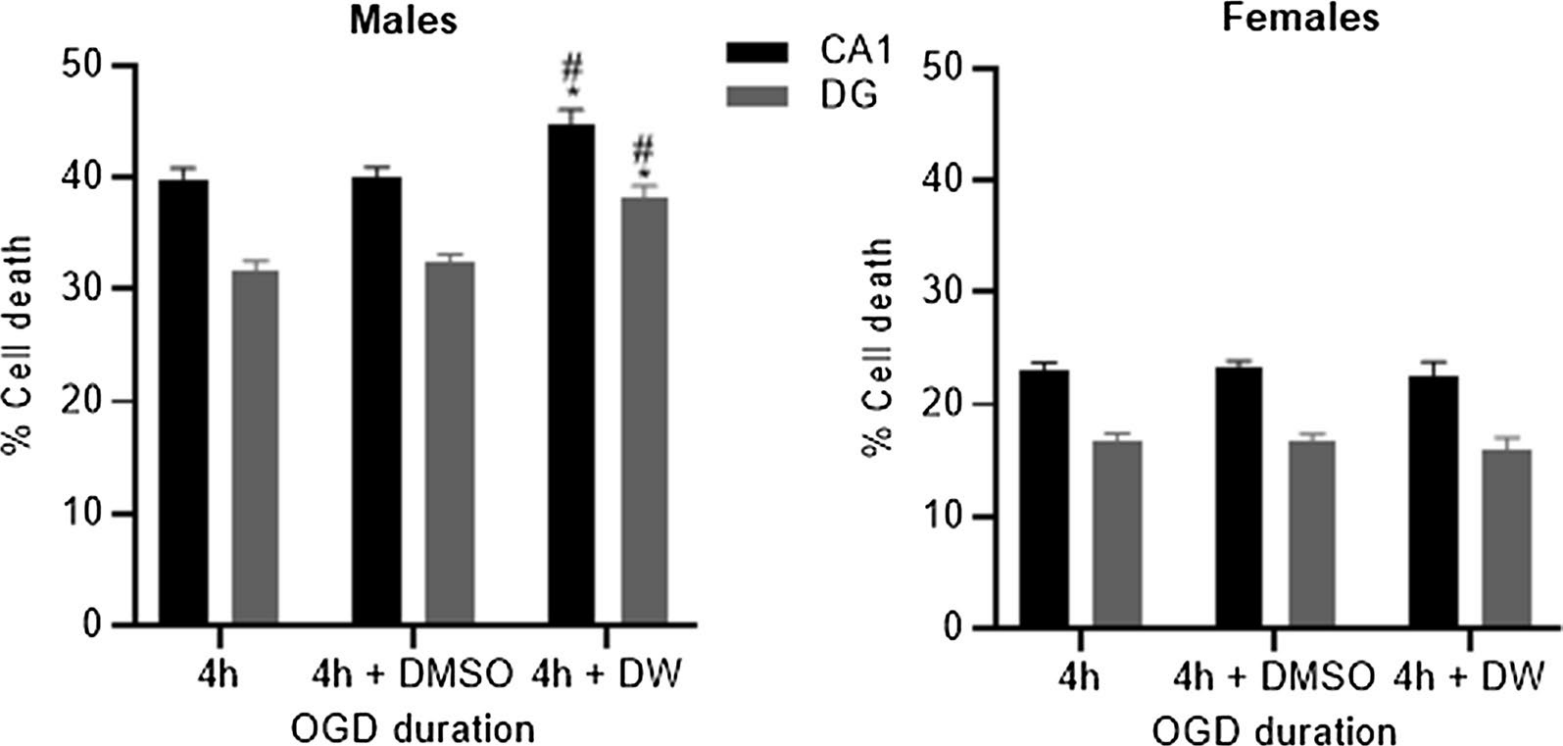
Cell death was analysed in both the CA1 and dentate gyrus (DG) regions following OGD and inclusion of DMSO or distilled water (DW). In male-derived cultures, there was a significant increase in the amount of cell death, in both regions, following inclusion of DW (*P < 0.05 vs. OGD only; ^#^P < 0.05 vs. OGD + DMSO). In female-derived cultures there was no significant differences in the amount of cell death between the treatment conditions. Data are expressed as mean ± SEM and n = 8–18 independent wells

We then determined whether the effect of the various vehicle treatments (i.e. dimethyl sulfoxide, DMSO; distilled water) had any effect on the amount of cell death seen in the CA1 and DG regions (Fig. 2). In male-derived cells there was an increase in the amount of cell death following exposure to OGD and distilled water in comparison to OGD only or OGD and DMSO in the CA1 (P < 0.05) and dentate gyrus (P < 0.01) regions. In female-derived slices, the addition of either DMSO or distilled water had no effect on the amount of cell death in comparison to OGD only.

**Fig. 3:**
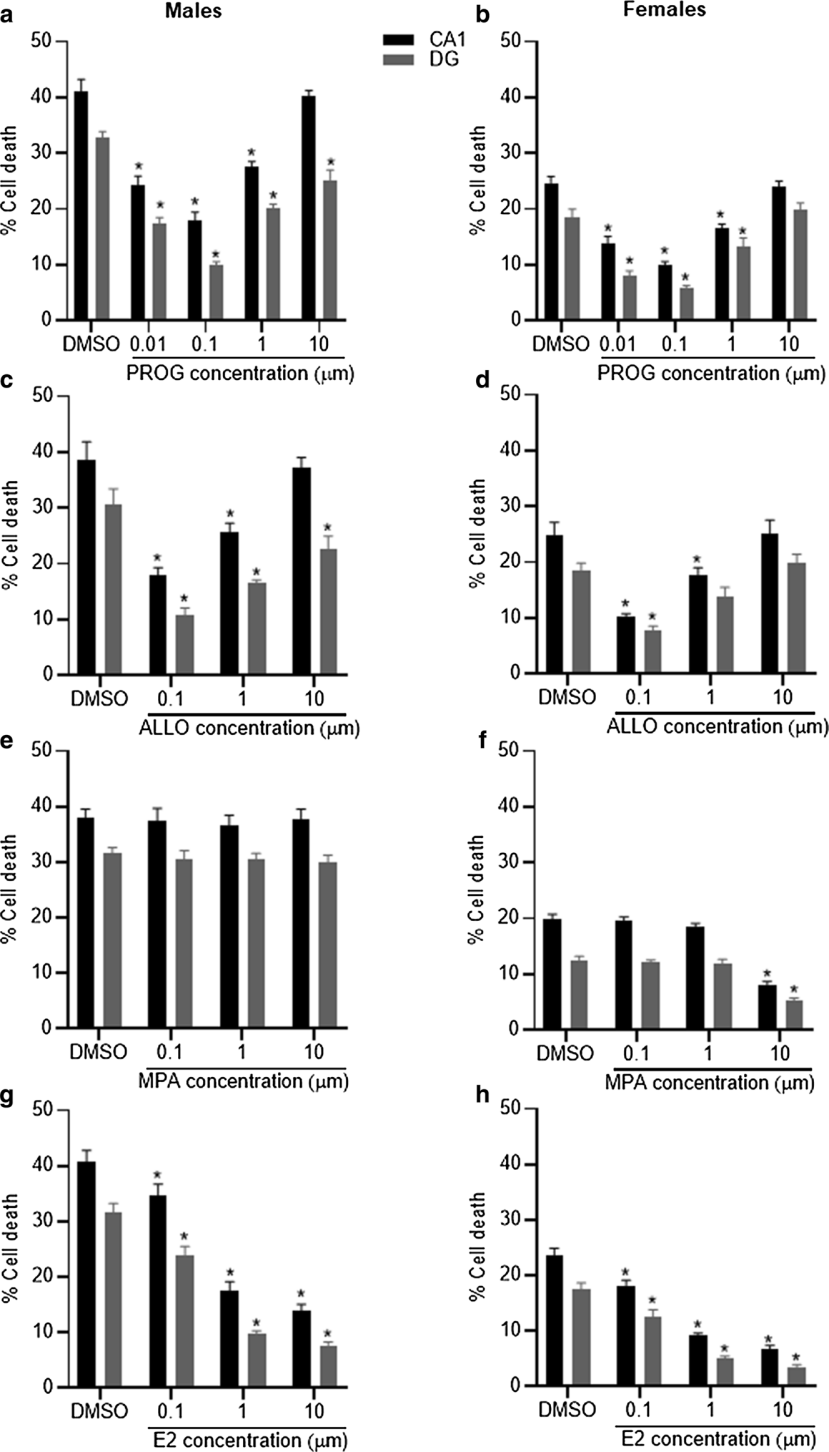
Cell death was analysed in both the CA1 and DG regions following OGD and exposure to varying concentrations of progesterone (PROG; **a**, **b**), allopregnanolone (ALLO; **c**, **d**), medroxyprogesterone acetate (MPA; **e**, **f**) and estradiol (E2; **g**, **h**). Data are shown separately for male (**a**, **c**, **e**, **g**) and female-derived (**b**, **d**, **f**, **h**) cultures. Significant (P < 0.05) reductions in the amount of cell death following hormone treatment compared to DMSO are indicated by *. Data are expressed as mean ± SEM and n = 8 independent wells

In order to determine the sex-specific effects of various steroid hormones on the amount of cell death following OGD we exposed cultures to progesterone, allopregnanolone, medoxyprogesterone or estradiol at various concentrations. Following treatment with progesterone, a one-way ANOVA revealed a significant reduction in the amount of cell death in male-derived slices in the CA1 (F_5,40_ = 47.68, P < 0.001) and DG (F_5,40_ = 58.78, P < 0.001) regions and in female-derived slices in the CA1 (F_5,40_ = 44.55, P < 0.001) and DG (F_5,40_ = 28.34, P < 0.001) regions (Fig. 3a, b). Post-hoc tests showed that progesterone significantly (P < 0.001) reduced cell death at all concentrations tested, apart from 10 µm in female-derived slices and 10 µm in the CA1 region in male-derived slices. Allopregnanolone treatment, analysed via one-way ANOVA, revealed a significant reduction in the amount of cell death in both the CA1 and DG regions in both male (CA1 F_4,32_ = 21.23, P < 0.001; DG F_4,32_ = 18.98, P < 0.001) and female-derived (CA1 F_4,32_ = 14.80, P < 0.001; DG F_4,32_ = 15.50, P < 0.001) slices (Fig. 3c, d). Post-hoc tests showed that allopregnanolone significantly (P < 0.001) reduced cell death at 0.1 and 1.0 µm in the CA1 region in both male and female-derived cells. Within the DG region, allopregnanolone significantly reduced the amount of cell death at all concentrations tested in male-derived slices but was only effective at 0.1 µm in females. There was no significant effect of MPA treatment in male-derived cells in either the CA1 (P = 0.95) or DG (P = 0.85) regions. In female-derived slices, MPA did significantly reduce the amount of cell death in the CA1 (F_4,32_ = 64.12, P < 0.001) and DG (F_4,32_ = 39.10, P < 0.001) regions (Fig. 3e, f). Post-hoc tests showed that, in female-derived slices, cell death was only significantly reduced (P < 0.001) in the presence of 10 µm MPA in both CA1 and DG regions. Following estradiol treatment, a one-way ANOVA revealed a significant reduction in the amount of cell death in both the CA1 and DG regions in both male (CA1 F_4,32_ = 55.98, P < 0.001; DG F_4,32_ = 92.40, P < 0.001) and female-derived (CA1 F_4,32_ = 83.22, P < 0.001; DG F_4,32_ = 46.11 P < 0.001) slices (Fig. 3g, h). Post-hoc tests showed that estradiol significantly (P < 0.05) reduced cell death at all concentrations tested.

**Fig. 4:**
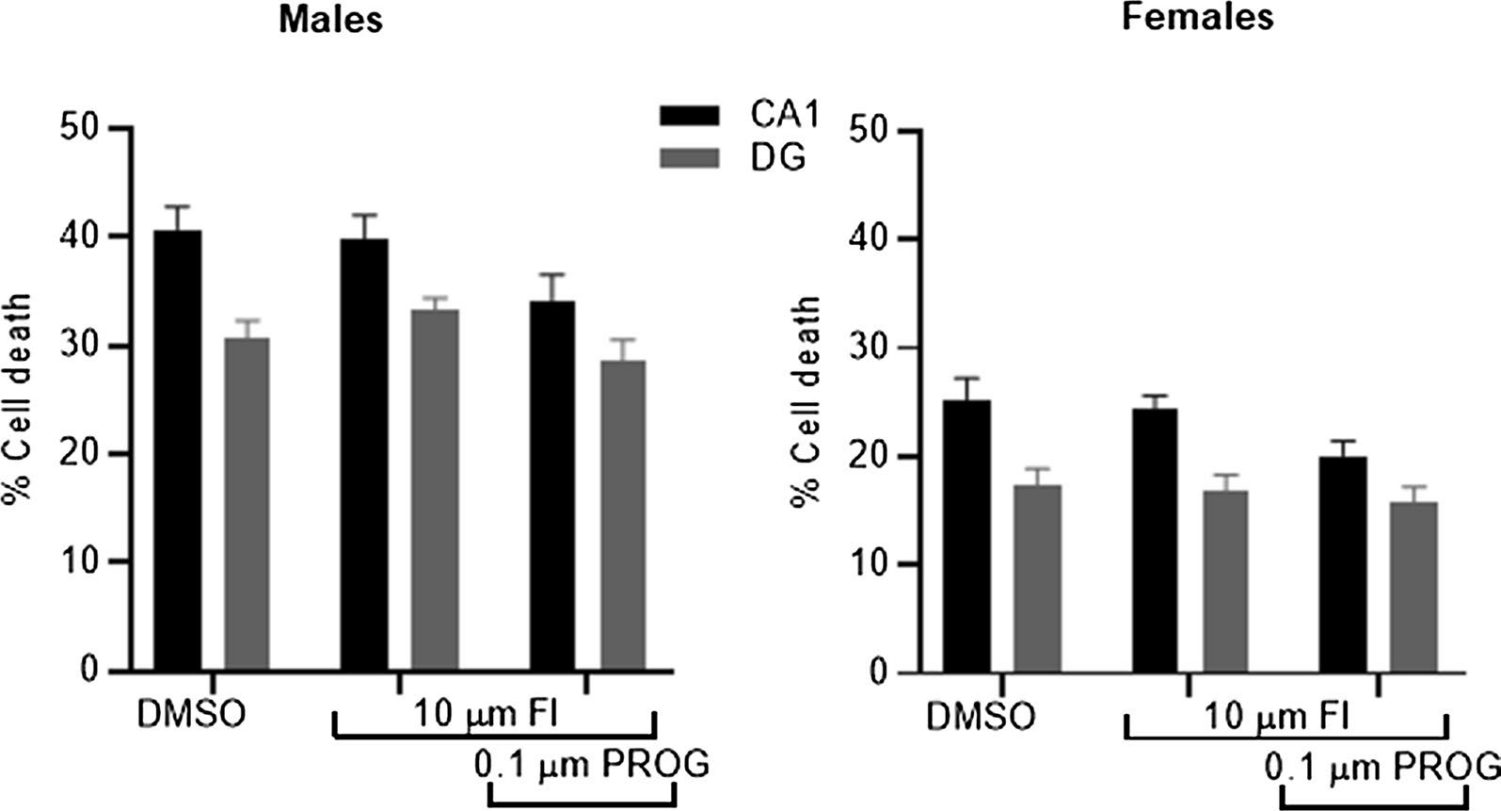
Cell death was analysed in both the CA1 and DG regions following OGD and exposure to finasteride (FI) or FIe and progesterone. Finasteride did not affect the amount of cell death in female and male-derived cultures. The presence of FI with progesterone prevented the reduction in cell death seen with progesterone only treatment (*P < 0.05 vs. DMSO). Data are expressed as mean ± SEM and n = 8 independent wells

In order to test the hypothesis that progesterone is neuroprotective via its conversion to the active metabolite allopregnanolone we applied progesterone, at a dose reported above to be protective, in conjunction with 10 µm finasteride. Finasteride, a 5α-reductase inhibitor, prevents the conversion of progesterone to allopregnanolone. A one-way ANOVA revealed, that in both male and female-derived slices, there was no significant change in the amount of cell death seen in the CA1 (males, P = 0.09; females, P = 0.05) and DG (males, P = 0.14; females, P = 0.71) regions in the presence of finasteride or finasteride in combination with 0.1 µm progesterone in comparison to DMSO-only (Fig. 4). Thus, finasteride-only did not affect the amount of cell death and the protective effect we had seen previously with 0.1 µm progesterone was absent in the presence of finasteride.

**Fig. 5:**
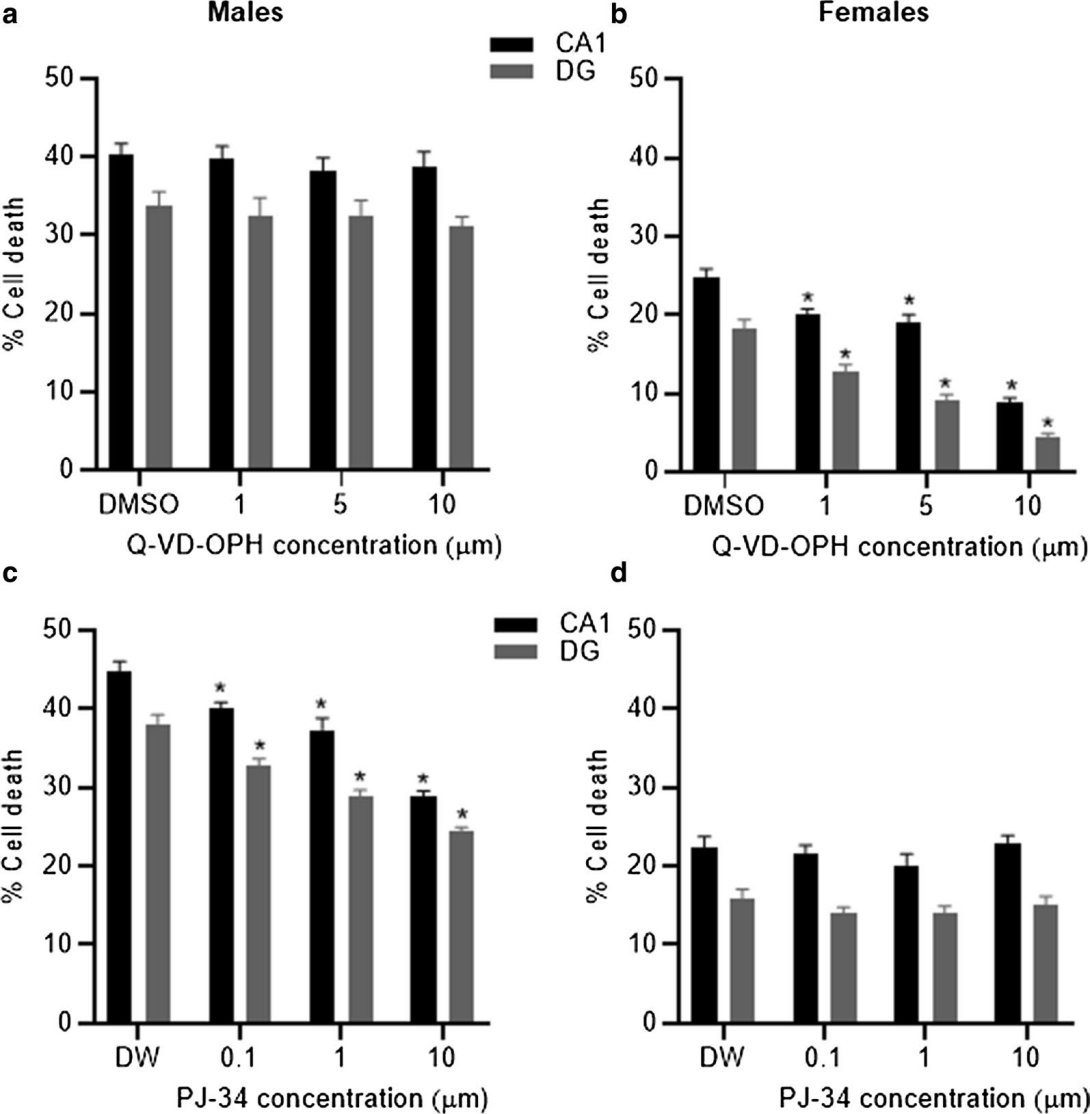
The addition of Q-VD-OPH did not affect the amount of cell death seen in male-derived slices (**a**). Exposure to Q-VD-OPH, at all concentrations tested, significantly reduced the amount of cell death in CA1 and DG regions compared to DMSO under OGD conditions in female-derived slices only (**b**, *P < 0.05 vs. DMSO). Exposure to PJ-34 did not affect the amount of cell death seen in female-derived slices (**c**). Treatment with PJ-34, at all concentrations tested, significantly reduced the amount of cell death in CA1 and DG regions compared to distilled water (DW) in male-derived slices only (**d**, *P < 0.05 vs. DW). Data are expressed as mean ± SEM and n = 8 independent wells

We went on to determine if there were any sex-specific effects in the efficacy of inhibitors of caspase (i.e. QJ-VD-OPH) and PARP (i.e. PJ-34) activity at reducing cell death. Following treatment with QJ-VD-OPH, a one-way ANOVA revealed no significant difference in the amount of cell death in both the CA1 (P = 0.83) and DG (P = 0.8) regions in male-derived slices (Fig. 5a). In female-derived slices, a one-way ANOVA revealed that treatment with QJ-VD-OPH significantly reduced the amount of cell death in both the CA1 (F_4,32_ = 49.8, P < 0.001) and DG (F_4,32_ = 46.58, P < 0.001) regions (Fig. 5b). Post-hoc tests showed that, in female-derived slices, QJ-VD-OPH significantly (P < 0.01) reduced cell death at all concentrations tested. Following treatment with PJ-32, a one-way ANOVA revealed a significant reduction in the amount of cell death in both the CA1 (F_4,32_ = 37.73, P < 0.001) and DG (F_4,32_ = 47.31, P < 0.001) regions in male-derived slices (Fig. 5c). Post-hoc tests showed that, in male-derived slices, PJ-32 significantly (P < 0.05) reduced cell death at all concentrations tested. In female-derived slices, a one-way ANOVA revealed that treatment with PJ-32 had no significant effect on the amount of cell death in the CA1 (P = 0.39) and DG (P = 0.41) regions (Fig. 5d).

**Fig. 6:**
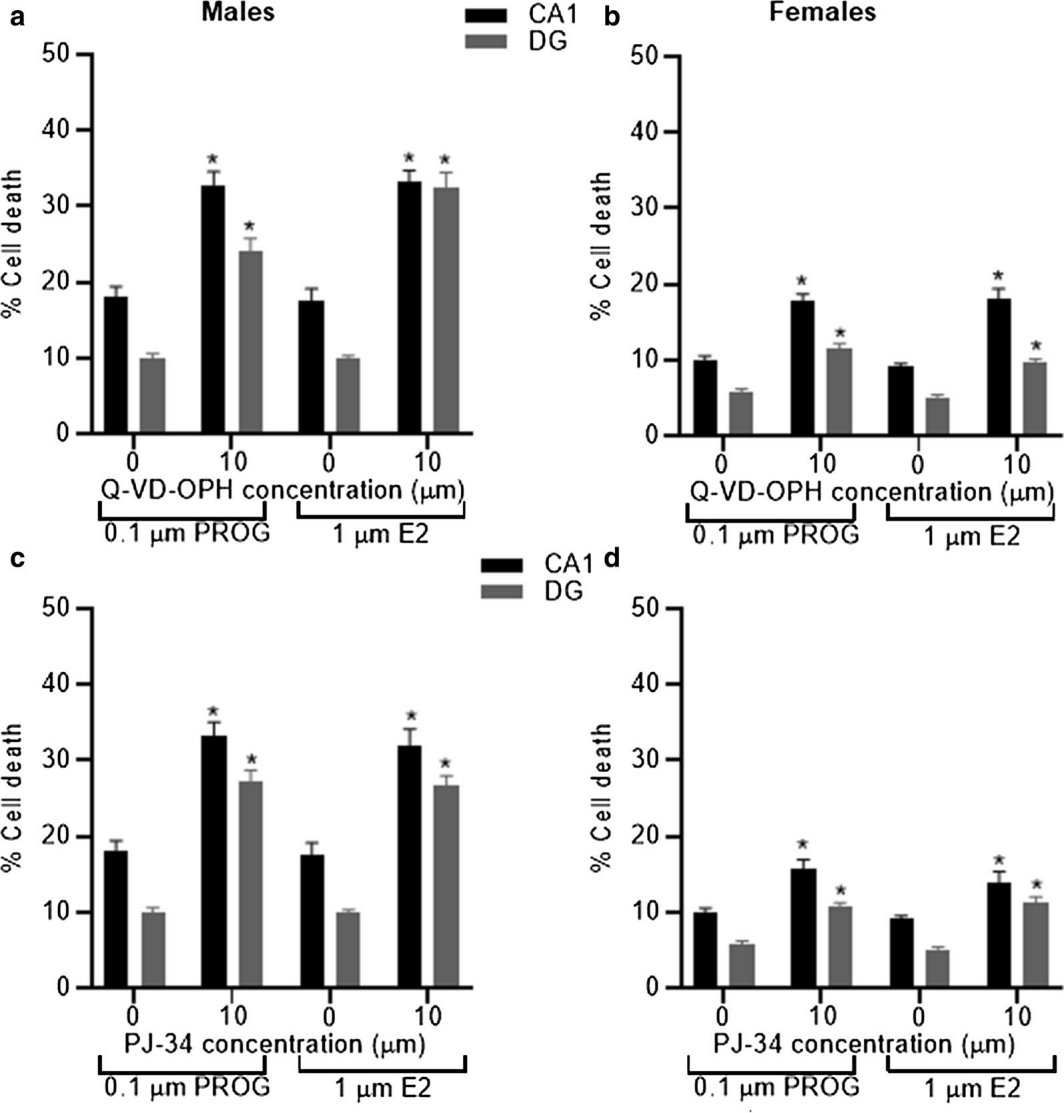
The presence of Q-VD-OPH, in both male and female-derived slices, in combination with either progesterone or estradiol significantly increased (*P < 0.01) the amount of cell death seen in comparison to progesterone or estradiol only in both CA1 and DG regions (**a**, **b**). The addition of PJ-34, in both male and female-derived slices, in combination with either progesterone or estradiol significantly increased (*P < 0.01) the amount of cell death seen in comparison to progesterone or estradiol only in both CA1 and DG regions (**c**, **d**). *PROG*: progesterone, E2: estradiol; data are expressed as mean ± SEM and n = 8 independent wells

Finally, we aimed to determine if the protection seen with progesterone and estradiol was still present in the presence of inhibitors of specific elements of the apoptotic pathways. We tested progesterone and estradiol in the presence or absence of a caspase inhibitor, Q-VD-OPH, or a PARP inhibitor, PJ-34. The addition of Q-VD-OPH, in both male and female-derived slices, to progesterone or estradiol at a concentration previously shown above to be protective, significantly (P < 0.001) increased the amount of cell death in comparison to progesterone- or estradiol-only in both CA1 and DG regions (Fig. 6). Likewise, the addition of PJ-34, in both male and female-derived slices, to progesterone or estradiol at a concentration previously shown (see above) to be protective significantly (P < 0.001) increased the amount of cell death in comparison to progesterone- or estradiol-only in both CA1 and DG regions (Fig. 6). Thus, suggesting that part of the protective effects of progesterone and estradiol were lost in the presence of either a caspase inhibitor, Q-VD-OPH, or a PARP inhibitor, PJ-34, in both sexes.

## Discussion

The aim of this study was to determine if sexual dimorphism occurs in the protective effects of steroid hormones and the mechanisms of cell death activated following ischemia. We observed that progesterone, allopregnanolone and estradiol were protective in terms of reducing the amount of cell death in both male- and female-derived tissue whereas medoxyprogesterone acetate was only protective in female-derived tissue. We also showed that caspase inhibition reduced cell death in female-derived tissue whereas PARP inhibition reduced cell death in male-derived tissue revealing differences between the sexes in the importance and contribution of various elements of the cell death pathways to the damage produced following an ischemic insult.

Here we utilised a sex-specific in vitro model of cell death which allows sex differences to be investigated in terms of responsiveness to CNS injury and treatments. It is becoming increasingly clear that sex differences occur in terms of the incidence, pathology and response to treatment for a number of CNS disorders [25]. In terms of ischemia, although in vivo models may be more representative of human stroke, in vitro models do offer certain advantages as they allow investigation of both treatment effects and pathological mechanisms under a controlled environment avoiding possible confounding effects of temperature and vascular components [26]. In vitro models generally use neonatal tissue and therefore may be of limited relevance to the adult-ageing brain, in which stroke is more prevalent. Although data from preclinical studies and pediatric populations do demonstrate that sex differences do exist within the developing brain in the response to stroke [27]. The use of sex-typed cells, as described here, means that any difference in outcome observed i.e. cell death, occurs as a consequence of inherent sex differences within the cells, from prenatal hormone exposure, or a combination of the two. However, neonatal and pre-adolescent cell populations have limited exposure to circulating sex steroid hormones thus observed sex differences are probably independent of hormonal activational effects. In our cell populations, any differences between the sexes are probably accounted for by intrinsic sex differences related to the sex chromosomes rather than organizational effects of prenatal sex hormone exposure. Here we confirmed that sex differences occurred in the amount of cell death following OGD with male-derived cells being more sensitive to OGD-induced cell death than female-derived cells. This is consistent with clinical and in vivo studies which show, for example, that younger female rodents are more resistant to ischemic brain damages than younger male rodents [28].

In terms of steroid hormones, progesterone, allopregnanolone and estradiol were protective, in terms of reducing the amount of cell death, in tissue derived from both sexes. Previous in vitro and in vivo studies have reported the protective effects of progesterone and estradiol but it is interesting here to determine their sex-specific effects. Progesterone and its active metabolite, allopregnanolone, are reported to be neuroprotective via a variety of mechanisms [29]. To test the hypothesis that progesterones effects are largely conveyed via its conversion to allopregnanolone we applied it in the presence of a 5α-reductase inhibitor, finasteride, which prevented the protective effects of progesterone. Thus, in this experimental set up it would appear that progesterones protective effects are mediated via conversion to an active metabolite, such as allopregnanolone. However, progesterone is firstly converted to 5α-dihydroprogesterone (DHP) and then allopregnanolone and these conversions are catalysed by 5α-reductase and 3α-hydroxysteroid dehydrogenase enzymes, respectively. The metabolites may have differing effects under ischemic conditions as, for example, DHP, like progesterone, is able to act at the progesterone receptor whereas allopregnanolone acts at the GABA-A receptor. Finasteride inhibits the synthesis of 5α-reduced neurosteroids and previous in vitro studies using mixed cell cultures have reported inhibition of progesterone protection in the presence of finasteride [30, 31]. However, it may be worthy to investigate other metabolites of progesterone as they have been shown to play a role in the protective properties of endogenous progesterone following experimental stroke [32]. MPA, is a synthetic progestin used commonly as part of hormonal replacement therapy or the contraceptive pill. In this study, MPA was found to be protective in female-derived tissue but had no effect in males whereas others have suggested that MPA is not able to provide protection of CNS tissue when utilising mixed cell cultures [33]. Sex differences in the protective effects of MPA may be due to its ability to bind not only to the classical progesterone receptor but also has have stimulator or inhibitory actions at glucocorticoid, androgenic or mineralocorticoid receptors which have shown to differ in their expression between the sexes [34].

We provide further evidence here that different elements of the cell death pathway, activated under ischemic conditions, differ between the sexes. The pan caspase inhibitor, Q-VD-OPH, used in this study was only protective in female-derived tissue which is similar to results reported for another pan caspase inhibitor i.e. z-VADfmk [22]. Application of PJ34 to inhibit PARP was only protective in male-derived tissue which has been reported in vivo previously [35]. Thus, this study adds further evidence that male-derived tissue is more sensitive to caspase-independent cell death whereas female-derived tissue is more sensitive to caspase-dependent cell death. This has important implications for the design of appropriate treatments following ischemic stroke in terms of applicability to both sexes [36, 37]. However, as the neuroprotective effects of progesterone and estradiol were maintained in the presence of either a caspase or PARP inhibitor this would suggest that activation of either of these is not critical for the protective effects of progesterone or estradiol.

## Conclusions

Sex differences exist in both the amount of cell death produced and those elements of the cell death pathway activated following an ischemic insult. There are also some sex differences in the effectiveness of steroid hormones to provide neuroprotection following an ischemic insult. Thus, there is increasing evidence that sex must be taken into account when investigating future drug targets for CNS disorders, such as ischemic stroke.

## Methods

### Animals

In vitro cultures were prepared, as previously described [38, 39], from 4–9 days old mouse C57/Bl6 pups housed in a specific pathogen free (SPF) unit with ad libitum access to food and water. Animals, typically weighing less than 10 g, were euthanised using humane cervical dislocation under UK Home Office regulations. The animal welfare and ethics committee of the University of Leicester approved all experimental protocols. All animals were supplied by Charles River UK. Sex of the pups was determined by visible inspection of anogenital distance as female mice have a genital area much closer to the anus compared to male mice. Also, pigmented cells on the scrotum are visible to the naked eye on the day of birth in male mice compared to female mice in C57/Bl6 pups and female mice have 10 nipples compared to male mice that do not have nipples [40].

### In vitro ischemia

Organotypic hippocampal cultures (OHSCs) were prepared according to the methods of Stoppini et al. [41] with some modifications, as we have reported previously [38, 39]. Briefly, brains were removed from the animals and the hippocampi dissected. The hippocampi were then sliced at 350 µm using a McIlwain tissue chopper and prepared in ice-cold dissecting medium containing HBSS (Hanks Balanced Salt Solution), 4.5 mg/ml glucose solution and 3.75 µg/ml amphotericin B. The slices were separated and placed onto Millicell membrane inserts (0.4 µm, Millipore) in six well plates, and cultured in growth medium containing 50% MEM (minimal essential medium), 25% horse serum, 25% HBSS, 0.5 mM glutamine, 4.5 mg/ml glucose and 3.75 µg/ml amphotericin B. Cultures were maintained in a humidified incubator with 5% CO_2_ at 37 °C for 14 days and culture medium was changed every 3 days. All substances used for preparation and maintenance of cultures were obtained from Sigma unless stated.

OHSCs were exposed to OGD at day 14 by placing in OGD medium containing 75% MEM, 25% HBSS, 1 mM glutamine and 3.75 µg/ml amphotericin B which was bubbled for 30 min with 5% CO_2_ and 95% N_2_. After two washes with the OGD medium, 1 ml of OGD medium was placed in the well and plates were transferred to an anoxic chamber. The chamber was sealed and pumped with 5% CO_2_ and 95% N_2_ for 10 min then placed in an incubator at 37 °C for 4 h (OGD duration). The cultures were returned to oxygenated serum-free culture medium and placed back in the incubator for a further 24 h.

### Drug treatments

Following OGD exposure, the cultures were returned to oxygenated serum-free culture medium containing one of the following treatments for a further 24 h: culture medium only, DMSO only, distilled water (DW) only, progesterone (0.01, 1.0 and 10 µm in DMSO), allopregnanolone (0.1, 1.0 and 10 µm in DMSO), medroxyprogesterone acetate (0.1, 1.0 and 10 µm in DMSO), 17β-estradiol (0.1, 1.0 and 10 µm in DMSO), finasteride (10 µm in DMSO ± 0.1 µm progesterone), Q-VD-OPh (1, 5 and 10 µm in DMSO), or PJ-34 (0.1, 1.0 and 10 µm in DW).

### Assessing cell death

To allow quantification of cell death, 30 min before the termination of experiments, the fluorescent cell death marker propidium iodide (PI, 5 µg/ml) and Hoechst (5 µg/ml) were added to the medium. At the termination of experiments, slices were fixed with 4% paraformaldehyde at 4 °C for 2 h and then briefly washed in Phosphate Buffered Saline, removed from the membrane inserts, mounted onto glass slides in PBS and imaged using a Nikon epifluorescence microscope. As previously described [38, 39] for each hippocampal slice, images were taken, using a Nikon microscope, from two different regions (CA1 and DG). For each region, two photos were taken, one image showing PI-labelled cells and the other showing Hoescht-labelled nuclei. The number of cells in each image was counted manually by an individual blinded to the experimental condition. The percentage cell death was calculated by dividing the number of dying cells, as indicated by PI-labelling, by the total number of cell nuclei, as indicated by Hoescht immunoreactivity.

### Data analysis

Data are reported as means ± standard error of the mean (SEM) and data were normally distributed, as tested using the D’Agostino and Pearson normality test. Statistical significance between two conditions (e.g. normoxia vs. OGD, males vs. females) was determined using Student’s t-test, whereas, for comparison between more than two conditions (e.g. drug concentrations), statistical significance was calculated using one-way analysis of variance (ANOVA) followed by Tukey’s post hoc tests. The data were analysed using Graph Pad Prism Version 8.0 for Windows and the criterion for statistical significance is P < 0.05. Slice cultures were prepared from 2–4 pups (of each sex) and n is equal to the number of independent wells with each well having three slices.

## Data Availability

Dataset available on reasonable request from the corresponding author.

## Abbreviations

AIF: apoptosis-inducing factor
DG: dentate gyrus
DMSO: dimethyl sulfoxide
DW: distilled water
HBSS: Hanks Balanced Salt Solution
MPA: medroxyprogesterone acetate
MEM: minimal essential medium
OHSC: organotypic hippocampal cultures
OGD: oxygen and glucose deprivation
PARP: poly (ADP-ribose) polymerase
PI: propidium iodide
SEM: standard error of the mean
